# Prediction of visceral adipose tissue magnitude using a new model based on simple clinical measurements

**DOI:** 10.3389/fendo.2024.1411678

**Published:** 2024-07-10

**Authors:** Cundullah Torun, Handan Ankaralı, Lütfullah Caştur, Mehmet Uzunlulu, Ayşe Naciye Erbakan, Muhammet Mikdat Akbaş, Nesrin Gündüz, Mahmut Bilal Doğan, Müzeyyen Arslan Bahadır, Aytekin Oğuz

**Affiliations:** ^1^ Department of Internal Medicine, Istanbul Medeniyet University, Istanbul, Türkiye; ^2^ Department of Biostatistics and Medical Informatics, Istanbul Medeniyet University, Istanbul, Türkiye; ^3^ Department of Radiology, Istanbul Medeniyet University, Istanbul, Türkiye

**Keywords:** obesity, metabolic syndrome, cardiometabolic risk, visceral adipose tissue (VAT), multivariate analysis

## Abstract

**Aims:**

Waist circumference (WC) is a reliable obesity surrogate but may not distinguish between visceral and subcutaneous adipose tissue. Our aim was to develop a novel sex-specific model to estimate the magnitude of visceral adipose tissue measured by computed tomography (CT-VAT).

**Methods:**

The model was initially formulated through the integration of anthropometric measurements, laboratory data, and CT-VAT within a study group (n=185), utilizing the Multivariate Adaptive Regression Splines (MARS) methodology. Subsequently, its correlation with CT-VAT was examined in an external validation group (n=50). The accuracy of the new model in estimating increased CT-VAT (>130 cm^2^) was compared with WC, body mass index (BMI), waist-hip ratio (WHR), visceral adiposity index (VAI), a body shape index (ABSI), lipid accumulation product (LAP), body roundness index (BRI), and metabolic score for visceral fat (METS-VF) in the study group. Additionally, the new model’s accuracy in identifying metabolic syndrome was evaluated in our Metabolic Healthiness Discovery Cohort (n=430).

**Results:**

The new model comprised WC, gender, BMI, and hip circumference, providing the highest predictive accuracy in estimating increased CT-VAT in men (AUC of 0.96 ± 0.02), outperforming other indices. In women, the AUC was 0.94 ± 0.03, which was significantly higher than that of VAI, WHR, and ABSI but similar to WC, BMI, LAP, BRI, and METS-VF. It’s demonstrated high ability for identifying metabolic syndrome with an AUC of 0.76 ± 0.03 (p<0.001).

**Conclusion:**

The new model is a valuable indicator of CT-VAT, especially in men, and it exhibits a strong predictive capability for identifying metabolic syndrome.

## Introduction

Obesity is a significant risk factor for cardiometabolic diseases such as type 2 diabetes, hypertension, and coronary artery disease, and its prevalence has been on the rise over the years (1). Body mass index (BMI) has traditionally been utilized to assess obesity, with the World Health Organization defining a BMI above 30 kg/m² as indicative of obesity (2). While BMI is a useful predictor of general obesity, it does not provide information about the differentiation between adipose tissue and lean body mass, thus potentially failing to adequately reflect the cardiometabolic risk associated with obesity (3).

The importance of body fat distribution, rather than total adiposity, was first emphasized by Vague (4). Subcutaneous adipose tissue (SAT) is commonly distributed throughout the body, while visceral adipose tissue (VAT) is predominantly found in the abdominal region and is more strongly linked to adipocyte dysfunction compared to SAT (5, 6). Studies have shown that increased VAT is a significant risk factor for cardiometabolic diseases, whereas the relationship between SAT and cardiometabolic risk is less clear (7, 8). Thus, research on obesity has increasingly focused on visceral adiposity.

Magnetic resonance imaging (MRI), computed tomography (CT), and dual-energy x-ray (DXA) are considered the most accurate methods for assessing visceral adiposity. Single-slice MRI and CT scans have been shown to effectively represent the total volume of VAT (9, 10). However, the cost, limited accessibility, and inability to be routinely used for primary prevention have driven the search for simpler, more cost-effective, and reproducible methods.

Waist circumference (WC) and waist-to-hip ratio (WHR) are the most commonly employed methods for predicting an increased risk associated with visceral adiposity. Numerous studies have demonstrated that WC measurement is an effective method for predicting the risk associated with obesity, leading to its inclusion as one of the criteria for metabolic syndrome (11, 12). However, WC alone cannot distinguish between visceral and subcutaneous fat distribution.

To address this gap, Lemieux et al. developed the concept of “hypertriglyceridemic waist circumference” in 2000, associating triglycerides and WC with visceral adiposity (13). Building on this, novel indices like visceral adiposity index (VAI) and lipid accumulation product (LAP) were created (14, 15). In 2012, Krakauer et al. developed a body shape index (ABSI), and the following year, Thomas et al. introduced the body roundness index (BRI), which was superior to BMI in identifying metabolic syndrome (16, 17). Although these indices estimate the risk associated with visceral adiposity, none were designed to predict VAT amounts. The metabolic score for visceral fat (METS-VF), created using nonlinear fits of insulin resistance (METS-IR), waist-to-height ratio (WHtR), age, and sex, appears successful in quantifying VAT (18, 19). However, cutoff values for anthropometric indices predicting cardiometabolic risk vary by age, gender, and ethnicity, making it essential to validate these equations in different groups or develop new models (20). Multivariate Adaptive Regression Splines (MARS) is a machine learning algorithm designed for multivariate nonparametric and non-linear regression problems (21). It adapts to the data to capture relationships in the dataset and make predictions, creating a flexible and adaptive model.

This study aimed to develop a novel anthropometric-based model using MARS for predicting visceral adiposity in a Turkish population without diabetes and to evaluate the accuracy of this model in predicting visceral adipose tissue measured by computed tomography (CT-VAT) by comparing it to adiposity indices in use.

## Subject and methods

This study was conducted between January 2022 and November 2023 at a tertiary training and research hospital and received approval from the Ethics Committee of Istanbul Medeniyet University Göztepe Training and Research Hospital (Ethics Committee Number: 2021/0464).

### Study design and participants

#### Study group

The study group included patients aged 20-50, who had visited our hospital between January 2022 and November 2022 for any reason, received outpatient diagnosis or treatment, and had undergone abdominal CT scans, including the L3 level. The retrospective screening process for eligible patients occurred every two weeks over one year through the hospital’s electronic database, resulting in the examination of a total of 744 patient files.

**Figure 1:**
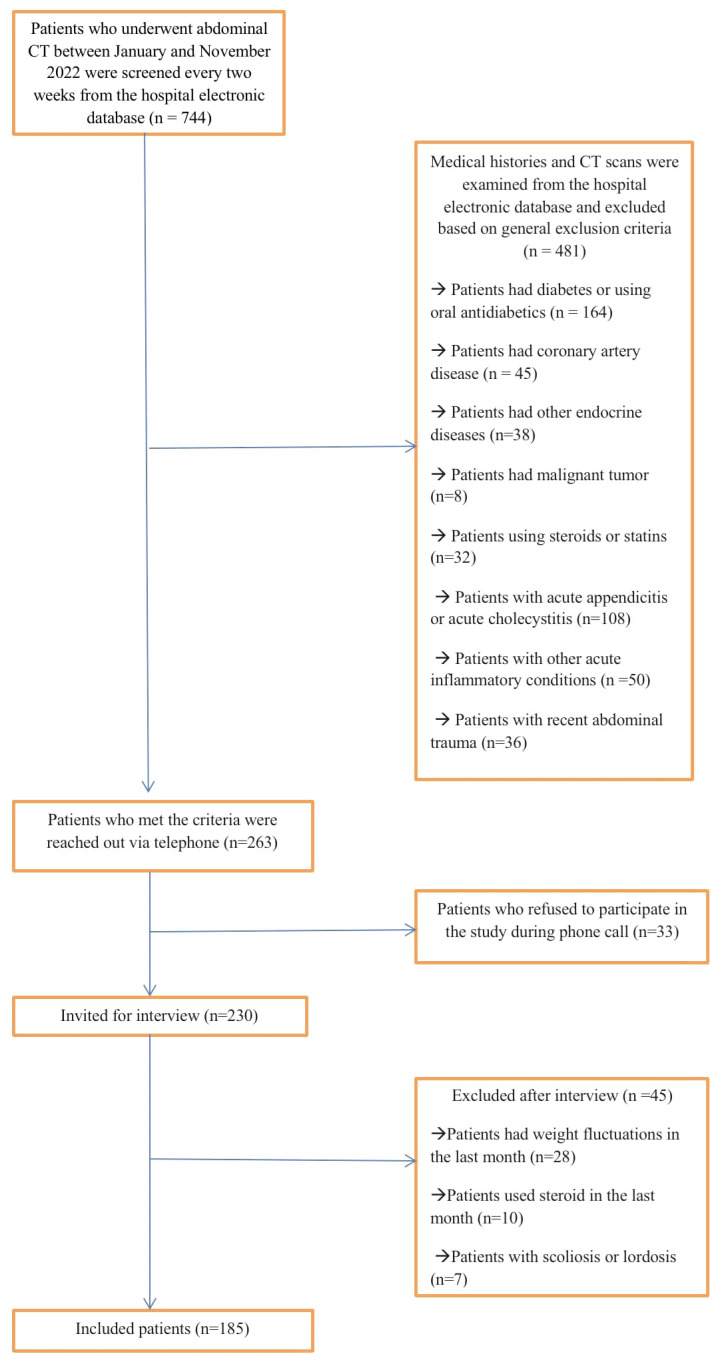
Flowchart depicting the process of inclusion and exclusion for the study group.

Patients with a history of malignancies, diabetes, coronary artery disease, chronic inflammatory diseases, those currently using steroids or antihyperlipidemic drugs, and those showing signs of acute inflammation on their CT scans were excluded from the study. Subsequently, the remaining patients (n=263) were contacted and invited to participate. Those who provided written consent were surveyed regarding their sociodemographic information, personal and family medical history, and smoking habits. A total of 185 eligible participants were ultimately enrolled in the study (**Figure 1**). Anthropometric measurements and the collection of blood samples took place within a maximum of one week from the screening day. This ensured that the time interval between obtaining CT scans and collecting blood samples, as well as conducting anthropometric measurements, did not exceed three weeks for any participant.

Blood samples were collected after an overnight fast of 8-12 hours including plasma glucose, triglycerides, high density lipoprotein cholesterol (HDL-C), and low-density lipoprotein cholesterol (LDL-C). Anthropometric measurements included height, weight, WC, hip circumference (HC), and neck circumference. Visceral and subcutaneous adipose tissue measurements were carried out by two radiologists utilizing the Horos v3.3.6 medical image software (https://horosproject.org/). After determining the level of the L3 vertebrae from sagittal sections, measurements were made on the axial section passing through this level.

Anthropometric measurements and laboratory data from patients in the study group were utilized for MARS predictions, leading to the development of a model to estimate CT-VAT values. Subsequently, internal validation was carried out using a 10-fold cross-validation approach within the same group.

#### External validation group

Upon completion of the model development process, the initiation of a re-screening process for external validation began. Between June and September 2023, files of patients aged 20-50, who underwent abdominal CT scans in the last two weeks, were screened biweekly from the hospital system. The same exclusion criteria were applied as in the study group, and patients meeting the criteria were consecutively invited to the hospital within a week for anthropometric measurements and blood sample collection.

The determination of sample size took into consideration the simple correlation between the predicted VAT from the new model and CT-VAT measurements. Assuming a moderate effect size (r=0.6), a Type-I error of 5%, and a Power of test of 90%, it was calculated that a total of 17 patients were needed. A total of 173 patient files were screened during this period. However, due to exclusion criteria from the study and inaccessibility, the final sample size was determined as 50.

Within this external validation group (n=50), an analysis of the correlation between the newly developed model and CT-VAT was conducted.

#### Metabolic healthiness discovery cohort

To evaluate the accuracy of the new model in identifying metabolic syndrome, data from a cohort of 616 individuals regularly followed up at our hospital were utilized. This cohort consisted of individuals with a BMI of 18 or higher who had visited the internal medicine outpatient clinics between January 2016 and December 2018 for various reasons. Their medical histories, along with data from blood tests, blood pressure measurements, and anthropometric measurements taken on the index date, were retrospectively examined. Following the exclusion of patients with diabetes and coronary artery disease, the data of 430 eligible patients were included in our study for analysis.

Patients were then categorized into groups with and without metabolic syndrome based on the criteria outlined in the National Cholesterol Education Program (NCEP) Adult Treatment Panel III (ATP III) (12).

### Model description and comparing with adiposity indices in use

#### Multivariate adaptive regression splines model

In this study, CT-VAT values were predicted with the help of the MARS model. The model does not have any assumptions and any type of variable can be included in the model. A special advantage of MARS lies in its ability to estimate contributions of some basic functions (BF) so that both additive and interactive effects of the predictors are allowed to determine the dependent variable. BFs are automatically selected to capture complex relationships of variables in the data set, and the number and locations of nodes are optimized to control the complexity of the model (22). The MARS model can also be used to evaluate the impact and importance of variables. It can also be used to control or exclude the influence of variables when making predictions. It seeks to achieve two objectives: an excellent fit to the data, and a simple model (21). In this study, the “earth” package of the R (ver.3.1.) program was used to develop the MARS model.

#### Evaluating the predictive accuracy of the MARS model for estimating visceral adiposity

The newly developed model’s predictive accuracy in estimating increased CT-VAT was compared with the below-defined adiposity indices (14–19).

The cutoff value for increased CT-VAT was set at 130 cm^2^, a value commonly used in the literature and recommended for Turkish individuals in the Turkey Adult Risk Factor Survey (TEKHARF) study (23).

Adiposity indices in use:


$$\begin{array}{l} \text{VAI in male }=\text{ WC }\left(\text{cm}\right)/\left(39.68+\left(1.88\times \text{BMI}\right)\right)\\ \times \left(\text{TG }\left(\text{mmol}/\text{l}\right)/1.03\right)\times \left(1.31/\text{HDL}–\text{C }\left(\text{mmol}/\text{l}\right)\right) \end{array}$$

$$\begin{array}{l} \text{VAI in female = WC }\left(\text{cm}\right)/\left(36.58+\left(1.89\times \text{BMI}\right)\right)\\ \times \left(\text{TG }\left(\text{mmol/l}\right)/0.81\right)\times \left(1.52/\text{HDL}–\text{C }\left(\text{mmol}/\text{l}\right)\right) \end{array}$$

LAP in male = (WC (cm)-65)×TG(mmol/l)

LAP in female = (WC (cm)-58) × TG (mmol/l)

$$\text{ABSI = WC }\left(\text{m}\right)/\left({\text{BMI}}^{2/3}{\times \text{ height}}^{1/2}\left(\text{cm}\right)\right)$$

$$\text{BRI}=364.2–365.5\times (1–{\left[\text{WC}/2\pi \right]}^{2}/{\left[0.5{\times \text{ height}}^{2}\right)}^{1/2}$$

$$\begin{array}{l} \text{METS}-\text{IR}=\text{ Ln}(\left(2\times \text{Fasting Glucose }\left(\text{mg/dl}\right)\right)\\ +\text{Tg }\left(\text{mg/dl}\right))\times \text{BMI})/\left(\text{Ln}\left(\text{HDL – C }\left(\text{mg/dl}\right)\right)\right) \end{array}$$

$$\begin{array}{l} \text{METS}-\text{VF}=\;4.466\;+\;0.011{\left(\text{Ln}\left(\text{METSIR}\right)\right)}^{3}\\ \;+\;3.239{\left(\text{Ln}\left(\text{WHtr}\right)\right)}^{3}\;+\;0.319\left(\text{Sex}\right)*\;+\;0.594\left(\text{Ln}\left(\text{Age}\right)\right) \end{array}$$

In this equations;

• VAI: Visceral adiposity index
• Tg: Triglyceride,
•HDL-C: High-density lipoprotein cholesterol
• LAP: Lipid accumulation product
• BRI: Body roundness index
• METS-IR: Metabolic score for insulin resistance
• WHtr: Waist (cm)/Height (cm)
• METS-VF: Metabolic score for visceral fat

*Sex is binary variable (male = 1, female = 0)

### Statistical analysis

Descriptive statistics of the variables were presented in tables as mean, standard deviation (SD), quartiles (25th, median, 75th), and frequencies (n, %). The normality assumption of numerical variables was tested with the Kolmogorov-Smirnov test and it was determined that they have a normal distribution. The success of the currently used indices and the newly developed model was examined in predicting increased CT-VAT using the ROC curves. DeLong et all (1988) approach was used for comparing of areas under correlated ROC curves (24). The ability of the newly developed model to identify patients with metabolic syndrome was assessed using the ROC curve, and the value with balanced and high sensitivity and specificity was chosen as the cut-off. A p-value less than 0.05 was accepted as the statistical significance. SPSS (ver. 23), **R** (ver. 4.2.2), Stata (ver. 14.1) and MedCalc (ver. 22.021) were used in the statistical analysis.

## Results

### Descriptive values

In the study group, we enrolled 185 participants, with a mean age of 38.2 ± 8 years and a female predominance (58.4%). The mean BMI for this group was 26.7 ± 5.7 kg/m², and 32 individuals (17.3%) exhibited metabolic syndrome. The external validation group included 50 participants with a mean age of 39.7 ± 8.8 years. The metabolic healthiness discovery cohort comprised 430 participants, with a mean age of 41.1 ± 12.2 years and a notable female predominance of 66.5%. Demographic, anthropometric, and laboratory characteristics of patients across the three groups are detailed in **Table 1**.

**Table 1:** Demographic, anthropometric, and laboratory characteristics of the study and validation groups.

	Study group (n=185)	External validation group (n=50)	Metabolic healthiness discovery cohort (n=430)
Female sex (%)	58.4	48	66.5
Metabolic syndrome (%)	17.3	30	26.5
Age (years)	38.2 ± 8	39.7 ± 8.8	41.1 ± 12.2
Waist circumference (cm)	89.5 ± 14.1	96 ± 13.5	95.5 ± 14.4
Hip circumference (cm)	104.0 ± 11.3	107.1 ± 12.5	110.2 ± 12.0
Body mass index (kg/m2)	26.7 ± 5.7	29.6 ± 5.9	30.9 ± 6.0
SBP (mmHg)	116.1 ± 14.5	124.1 ± 17	122 ± 16.3
DBP (mmHg)	79.9 ± 9.1	86.1 ± 10.9	83.8 ± 10
Fasting Glucose (mg/dL)	89.7 ± 11.3	89.4 ± 12.8	97.9 ± 22.4
LDL-Cholesterol (mg/dL)	110.8 ± 32.2	108.0 ± 28.2	129.3 ± 38.2
HDL-Cholesterol (mg/dL)	51.2 ± 13.4	48.0 ± 14.6	48.1 ± 13.2
Triglycerides (mg/dL)	114.1 ± 67.3	143.0 ± 91.6	135.2 ± 81.4
Waist-hip ratio	0.9 ± 0.1	0.9 ± 0.1	0.9 ± 0.1
METS-VF	6.3 ± 0.9	6.7 ± 0.7	6.7 ± 0.8
ABSI	0.1 ± 0.01	0.1 ± 0.0	0.1 ± 0.01
LAP	41.0 ± 39.3	61 ± 55.4	56.8 ± 46.0
BRI	4.3 ± 1.8	5.0 ± 1.9	5.2 ± 2.0
VAI	1.8 ± 1.4	2.3 ± 1.9	2.3 ± 1.8
SAT (cm²)	213.1 ± 117.3	240.8 ± 133.4	
CT-VAT (cm²)	135.0 ± 88.5	137.9 ± 72.4	

SBP, Systemic Blood Pressure; DBP, Diastolic Blood Pressure; METS-VF, Metabolic Score for Visceral Fat; ABSI, A Body Shape Index; LAP, Lipid Accumulation Product; BRI, Body Roundness Index; VAI, Visceral Adiposity Index; SAT, Subcutaneous Fat Area; CT-VAT, Visceral Adipose Tissue Measured by CT.

Data is presented in mean (± SD) unless otherwise stated.

The demographic and clinical characteristics of the study group were presented in **Table 2** with stratification by gender. Men displayed higher values in age, WC, WHR, systolic blood pressure, LDL-C, triglycerides, METS-VF, and ABSI, while women demonstrated higher levels of HDL-C and subcutaneous fat area (p< 0.05 for all).

**Table 2:** Demographic and clinical characteristics of participants in the study group.

	Male (n=77)	Female (n=108)	P value
Age	39.6 ± 7.3	37.3 ± 8.3	**0.048**
Waist circumference (cm)	93.6 ± 10.9	86.6 ± 15.5	**0.001**
Hip circumference (cm)	103.4 ± 7.6	104.5 ± 13.3	0.462
Fasting glucose (mg/dL)	88.6 ± 10.7	90.4 ± 11.8	0.306
SBP (mmHg)	122.3 ± 15.5	111.6 ± 12	**0.001**
DBP (mmHg)	80 ± 9.4	79.9 ± 9	0.953
LDL-Cholesterol (mg/dL)	116.9 ± 32.7	106.5 ± 31.3	**0.030**
HDL-Cholesterol (mg/dL)	45.4 ± 10.9	55.3 ± 13.5	**0.001**
Triglyceride (mg/dL)	131.8 ± 70.5	101.4 ± 62.3	**0.003**
Waist-hip ratio	0.9 ± 0.06	0.83 ± 0.07	**0.001**
Body mass index (kg/m^2^)	26.5 ± 4.2	27 ± 6.5	0.527
METS-VF	6.6 ± 0.7	6.1 ± 0.9	**0.001**
ABSI	0.08 ± 0.004	0.08 ± 0.006	**0.001**
LAP	46.2 ± 35.7	37.3 ± 41.5	0.128
BRI	4.3 ± 1.3	4.3 ± 2.1	0.809
VAI	1.9 ± 1.4	1.7 ± 1.4	0.286
SAT (cm^2^)	186.7 ± 91.5	232 ± 129.9	**0.006**
CT-VAT (cm^2^)	180.9 ± 89.9	102.3 ± 71.6	**0.001**

SBP, Systemic Blood Pressure; DBP, Diastolic Blood Pressure; METS-VF, Metabolic Score for Visceral Fat; ABSI, A Body Shape Index; LAP, Lipid Accumulation Product; BRI, Body Roundness Index; VAI, Visceral Adiposity Index; SAT, Subcutaneous Fat Area; CT-VAT, Visceral Adipose Tissue Measured by CT. P-values marked in bold indicate statistical significance.

### Modelling process

In the first stage, The MARS model included a total of nine predictors including age, gender, WC, HC, BMI, glucose, c-peptide, triglyceride, and HDL-C for the prediction of CT-VAT (cm^2^). We evaluated the total 20 BF including the main and their first-order interaction effects of these predictors in the model. Non-significant effects were removed from the model with the backward variable selection method, and 4 of the 9 variables were found to contribute significantly to the final model. The backward method was preferred over the forward method, as the forward method can result in suppressor effects. The final model produced by the backward only includes predictors that have a significant impact on the outcome variable. This allows for a more accurate and efficient model for making predictions.

Total of 7 basic functions, including the main and interaction effects of the 4 selected predictors, were included in the final model. When ranking the 4 selected variables by importance to the final model, WC, Gender, BMI, and HC stood out. The main and interaction effects, their coefficients, and the structure of BF, included in the final model were presented in **Table 3**. In the model, the constant term was estimated as 249. The model is as follows.

**Table 3:** Final MARS model for VAT prediction.

Basis Functions	Structure	Predicted VAT =	249 (Constant)
BF1	If gender “Female”		- 79.9
	Otherwise		0
BF2	If “WC< 111”		- 4.4*(111- WC)
	Otherwise		0
BF3	If “HC > 106”		- 5.14*(HC-106)
	Otherwise		0
BF4	If “BMI< 25.9”		- 29.5*(25.9-BMI)
	Otherwise		0
BF5	If “BMI > 25.9”		13*(BMI-25.9)
	Otherwise		0
BF6	If gender “Female” and “BMI< 29”		7.18*(29 - BMI)
	Otherwise		0
BF7	If “WC< 111” and “BMI< 28.4”		0.459*(111-WC)*(28.4-BMI)
	Otherwise		0

BF, Basis Functions in the Model; VAT, Visceral Adipose Tissue (cm²); WC, Waist Circumference (cm); BMI, Body Mass Index; HC, Hip Circumference (cm).

Predicted VAT = Constant + BF1+BF2+BF3+BF4+BF5+BF6+BF7

Predicted VAT = 249 - 79.9*Gender (Female)-4.4*max(0, 111 - WC)-5.14 * max(0, HC - 106)-29.5*max(0, 25.9 - BMI)+13*max(0,BMI - 25.9)+7.18*max(0, 29 -BMI)+0.459*(111-WC)*(28.4 - BMI)

For example, BF1 takes the value “-79.9” if the person’s gender is female and “0” if the gender of the person is male. BF2 takes the value “-4.4*(111-WC)” if WC<111, otherwise “0”. Other BFs are calculated similarly. Then, the CT-VAT value of that person is estimated by summing all the BF terms and the constant term.

A macro was created in Excel to easily perform model calculations. When the measured values are entered into the relevant sections of the macro, VAT values can be predicted.

The MARS model demonstrated an R-square (determination coefficient) of 78.15%, while WC, METS-VF, BMI, and WHR exhibited R-square values of 60.99%, 59.01%, 56.99%, and 48.44% respectively. Through an F-test comparing mean square errors, it was evident that the MARS model’s prediction accuracy significantly outperformed the others.

The goodness of fit criteria of the MARS model were presented in **Table 4**. RMSE and RRMSE in the table played an important role, especially in the comparison and selection of different models, and the model with the smaller value was selected. In addition, it was a preferred result that the other criteria in **Table 4** were small.

**Table 4:** Goodness-of-fit measures for MARS model.

Goodness-of-fit measures	Value
Root mean square error (RMSE)	41
Relative root mean square error (RRMSE)	31
Standard deviation ratio (SDR)	0.468
Coefficient of variation (CV)	30.6
Pearson’s correlation coefficients (PC)	0.884
Performance index (PI)	16
Mean error (ME)	0
Relative approximation error (RAE)	0.065
Mean relative approximation error (MRAE)	0.019
Mean absolute percentage error (MAPE)	33
Mean absolute deviation (MAD)	31
Akaike’s information criterion (AIC)	1392.5
Corrected Akaike’s information criterion (CAIC)	1393.3

After the model was developed, a 10-fold cross-validation approach was used to evaluate its internal validation, and it was observed that the internal validity was high.

### Predictive accuracy of the new model for identifying visceral adiposity in the study and external validation groups

**Figure 2:**
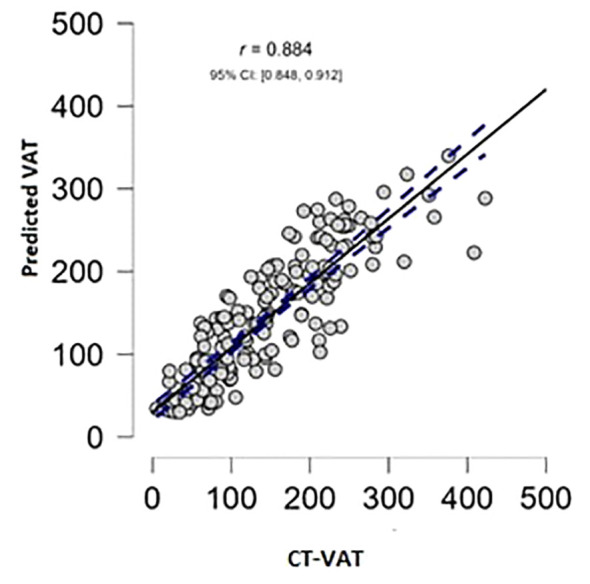
Association between predicted visceral adipose tissue (VAT) by the newly developed model and visceral adipose tissue measured by CT (CT-VAT) within the study group.

In the study group, a strong correlation (r=0.88) was observed between the new model and CT-VAT (**Figure 2**). The new model exhibited a sensitivity of 81.8% and specificity of 86.4% in predicting increased CT-VAT (>130 cm^2^) in males, while in females, the sensitivity was 96.9% with a specificity of 77.6%. New model demonstrated the highest predictive ability in identifying increased CT-VAT in males (AUC of 0.96 ± 0.02), outperforming all other indices (p<0.05 for all). In females, the AUC 0.94 ± 0.03, which is significantly higher than the VAI, WHR and ABSI, but similar to other indices (**Table 5**).

**Table 5:** Comparison of AUC values between new model and other indices for predicting increased CT-VAT in the study group.

Gender	Indices (AUC ± SE)	New model (0.957 ± 0.022) *P-values*
Male	BMI (0.907 ± 0.033)	**0.013**
	WC (0.922 ± 0.030)	**0.019**
	METS-VF (0.922 ± 0.030)	**0.020**
	VAI (0.731 ± 0.059)	**<0.001**
	WHR (0.889 ± 0.036)	**0.010**
	ABSI (0.702 ± 0.062)	**<0.001**
	LAP (0.876 ± 0.039)	**0.010**
	BRI (0.922 ± 0.030)	**0.020**
		New model (0.942 ± 0.030) *P-values*
Female	BMI (0.936 ± 0.031)	0.746
	WC (0.938 ± 0.031)	0.858
	METS-VF (0.939 ± 0.030)	0.887
	VAI (0.725 ± 0.057)	**<0.001**
	WHR (0.807 ± 0.050)	**0.003**
	ABSI (0.658 ± 0.060)	**<0.001**
	LAP (0.893 ± 0.039)	0.146
	BRI (0.925 ± 0.033)	0.372

BMI, Body Mass Index; WC, Waist Circumference METS-VF, Metabolic Score for Visceral Fat; VAI, Visceral Adiposity Index; WHR, Waist-hip Ratio; ABSI, A Body Shape Index; LAP, Lipid Accumulation Product; BRI, Body Roundness Index. P-values marked in bold indicate statistical significance.

**Figure 3:**
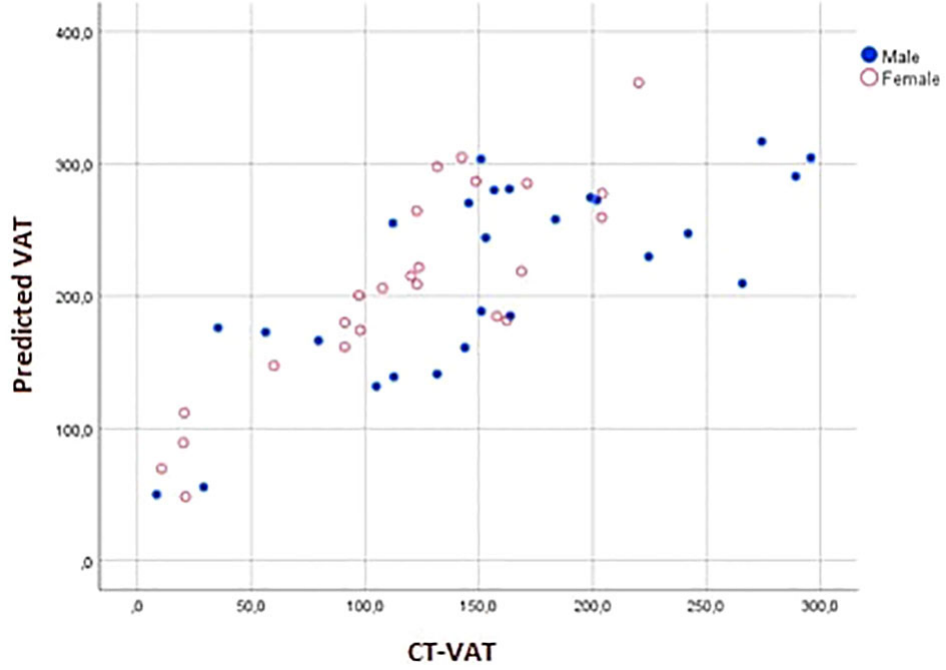
Association between predicted visceral adipose tissue (VAT) by the newly developed model and visceral adipose tissue measured by CT (CT-VAT) within the external validation group.

**Figure 4:**
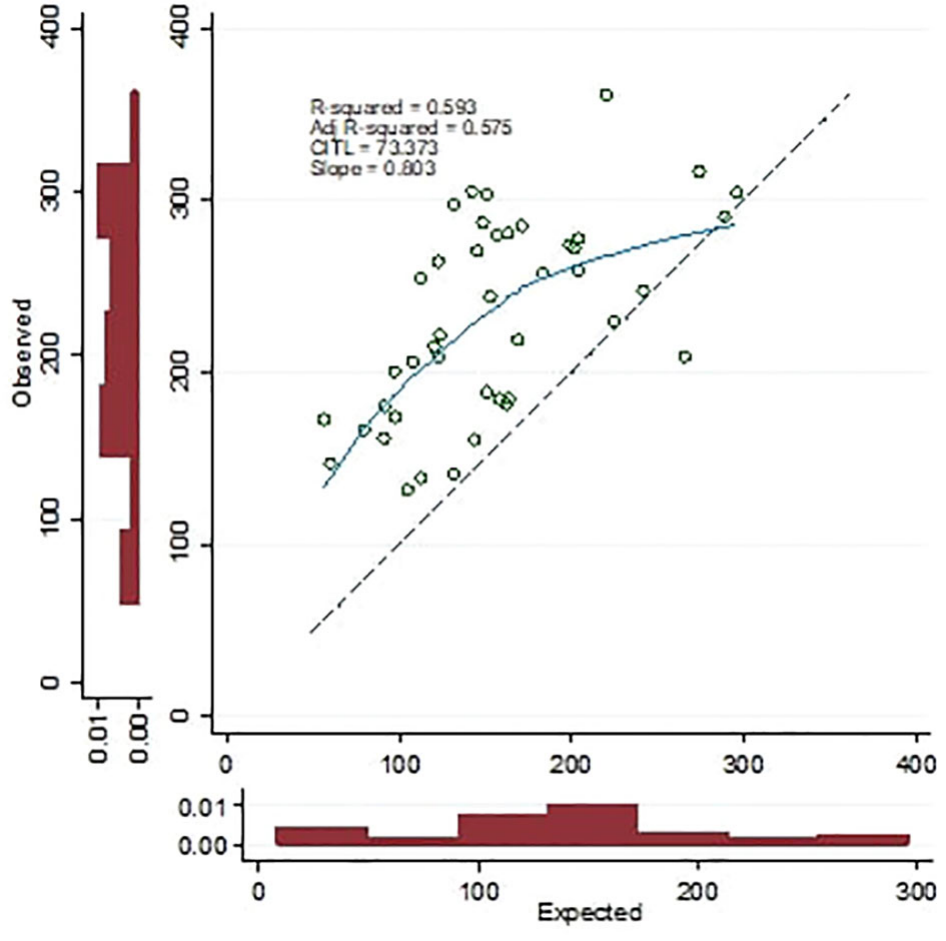
Calibration plot of the apparent performance of the new model in the study group.

In the external validation group, the new model demonstrated a statistically significant correlation of 0.775 with CT-VAT (P<0.001) (**Figure 3**). Based on calibration measures (R-square: 59.3%, CITL: 73.3, Calibration slope: 0.803), it was evident that the model exhibited strong performance (**Figure 4**). Notably, considerable prediction errors were observed for very low measured VAT values, yet predictions for medium to high values proved highly accurate. However, the model’s relative underperformance in cases with very low VAT values might not be critical for clinical practice; the primary concern lies in its efficacy in predicting VAT levels for individuals with medium to high values.

### Predictive accuracy of the new model in identifying metabolic syndrome

The diagnostic performance of the new model in identifying metabolic syndrome was evaluated. In the metabolic healthiness discovery cohort, using a cutoff value of 219.5, it demonstrated a sensitivity of 75.4% and specificity of 64.3%, with an AUC of 0.756 ± 0.030 (P<0.001). When the data of the patients from the study group (n=185), external validation group (n=50), and metabolic healthiness cohort (n=430) were collectively assessed (n=665), employing a threshold of 212.4, the new model showed a sensitivity of 78.3%, specificity of 65.5%, and an AUC of 0.781 ± 0.019 (P<0.001).

## Discussion

In this study, a strong correlation was observed between the newly developed model, utilizing gender, WC, BMI, and HC variables, and the amount of VAT measured by CT. The new model demonstrated superior predictive capability for increased CT-VAT (>130 cm^2^) in men compared to all other indices. In women, it outperformed WHR, ABSI, and VAI; however, its superiority over other indices did not reach statistical significance. Furthermore, it displayed high sensitivity and specificity in identifying metabolic syndrome at an appropriate cutoff value.

The significance of visceral adiposity as a major risk factor for cardiometabolic diseases highlights the importance of early detection. Studies comparing WC with other anthropometric measurements have demonstrated that WC alone is sufficient and practical in distinguishing abdominal obesity (25). However, the ability to quantitatively measure VAT using imaging methods has opened the door for developing new models that can add an additional impact to the predictive power of WC. In this study, we demonstrated that incorporating BMI and HC into a specific equation, alongside WC and gender, contributes to the prediction of VAT quantity, enhancing the predictive value of WC.

The use of BMI to predict the increased risk associated with obesity in epidemiological studies has been criticized (26). The EPIC study, examining the relationship between general obesity measured by BMI and abdominal obesity measured by WC with mortality, revealed a strong association between WC and WHR with mortality risk even after adjusting for BMI. This finding reinforces the notion that, when assessing the increased risk associated with obesity, WC holds an advantage over BMI (27). In some studies, the demonstration that reduced BMI is associated with increased mortality at a specific WC value has prompted the consideration of normalizing WC with BMI for predicting the increased risk associated with obesity (28, 29). Our study is noteworthy for demonstrating that an increase in BMI at a specific WC indicates an increase in visceral adiposity. Although the studies mentioned above indicating an inverse relationship between BMI and mortality may not have quantified visceral adiposity, they suggest that the relationship between mortality and anthropometric measurements is mediated through visceral adiposity. However, it should be noted that the patient population in these studies is predominantly composed of individuals with diabetes and/or coronary artery disease. To avoid bias, analyses of the association between adiposity and mortality should be limited to healthy individuals, given that preexisting diseases are inversely correlated with BMI and positively correlated with mortality (30). Indeed, studies conducted with individuals without diabetes and coronary artery disease have shown that BMI’s predictive accuracy for visceral adiposity is similar to WC, which is consistent with the results of our study (31, 32).

Studies have demonstrated that ABSI, based on the principle of normalizing WC with BMI, has a lower predictive value for predicting metabolic syndrome and diabetes development compared to WC and BMI (33, 34). Furthermore, in our study, ABSI showed weaker predictive capability for visceral adiposity in both genders compared to both BMI and WC. This suggests that normalizing WC with BMI might not be an appropriate method for predicting visceral adiposity and related conditions.

In the study group, the mean CT-VAT value was found to be lower in women compared to men, while the SAT value was higher. Upon examining the model, it is observed that the estimated VAT value at the same WC, BMI, and HP is lower in men. It is known that women have lower total VAT than men, and this is associated with the positive effects of estrogen on adipose tissue distribution and function in women (35, 36). In the literature, there is no consensus on gender-stratified WC cutoff values corresponding to increased visceral adiposity. Lemieux et al. found in their studies that a cutoff value of 95 cm is appropriate for increased VAT in both genders (37). In a study conducted in Korea, a WC cutoff value of 90 cm for men and 86 cm for women was identified, while in Japan, it was found to be 85 cm for men and 90 cm for women (38, 39). Considering the protective effects of estrogen on visceral adiposity in women, it is more plausible that men have more VAT than women at a specific WC, which is consistent with the findings of our study.

In our study, in both genders, WHR showed a lower association with CT-VAT compared to the new model. In the past, WHR was commonly employed in obesity assessments. However, studies highlighting the independent effectiveness of WC in relation to visceral adiposity led to a decreased emphasis on the clinical use of WHR (27). Although our study also demonstrates an inverse relationship between increasing HC at a specific WC value and visceral fat, interpreting this relationship as a simple numerator/denominator ratio may not be accurate. In clinical practice, it is important to recognize that both the numerator and denominator of WHR increase when they gain weight and decrease when they lose weight. Therefore, while hip measurement may be helpful in predicting visceral fat, relying solely on the simple waist/hip ratio can be misleading. It is necessary to take into account a more intricate relationship between these factors.

The strong relationship between the increase in visceral fat accumulation and insulin resistance is well established. Demonstrating the success of models developed to predict visceral fat in also identifying metabolic syndrome and type 2 diabetes enhances the reliability of such models. In our study, the finding that the newly developed model is successful in distinguishing metabolic syndrome in individuals without diabetes supports the clinical applicability of the model.

The study has several strengths. Firstly, the validation of the new model employed robust statistical methods. Secondly, the use of a small number of readily available covariates in the model enhances its generalizability to external datasets and routine clinical use. However, certain limitations should be acknowledged. Firstly, despite efforts to match risk factors, such as excluding patients with coronary artery disease and diabetes mellitus, the study did not consider patients’ smoking status and family history of cardiometabolic diseases. Secondly, the impact of menopausal status on visceral adipose tissue in women was not assessed. Although the menopausal status of patients was not queried, the inclusion criteria covering individuals between 20-50 years old and the mean age of women in the study group being 37.3 ± 8.3 suggest that this omission may not significantly affect the study results. Thirdly, the exclusion of patients with diabetes and those using antihyperlipidemic medication, which can alter glucose and triglyceride levels, used in the adiposity indices mentioned in the study and in the development of new formulas, caused a restriction in the study population. However, the presence of prediabetes and hyperlipidemia were not exclusion criteria. Additionally, most patients using antihyperlipidemic drugs were on statins for primary and secondary cardiovascular prevention, with relatively few taking these medications specifically for hypertriglyceridemia. Therefore, it can be said that the study group adequately represents patients with metabolic syndrome. Lastly, the single-center design of the study complicates the generalizability of the findings to the wider population.

In conclusion, it was found that the new model based on anthropometric measurements is successful in predicting visceral adiposity in apparently healthy individuals compared to current adiposity indices. These findings suggest that the practical use of this model may be beneficial for early identification of cardiometabolic risks associated with visceral adiposity in routine clinical settings.

## Data availability statement

The raw data supporting the conclusions of this article will be made available by the authors, without undue reservation.

## Ethics statement

The studies involving humans were approved by Ethics Committee of Istanbul Medeniyet University Göztepe Training and Research Hospital. The studies were conducted in accordance with the local legislation and institutional requirements. The participants provided their written informed consent to participate in this study.

## Author contributions

CT: Formal analysis, Investigation, Methodology, Writing – original draft, Writing – review & editing. HA: Methodology, Validation, Writing – original draft, Writing – review & editing. LC: Data curation, Investigation, Writing – original draft, Writing – review & editing. MU: Conceptualization, Investigation, Supervision, Writing – original draft, Writing – review & editing. AE: Formal analysis, Project administration, Writing – original draft, Writing – review & editing. MA: Data curation, Formal analysis, Software, Writing – original draft, Writing – review & editing. NG: Methodology, Software, Visualization, Writing – original draft, Writing – review & editing. MD: Conceptualization, Methodology, Software, Writing – original draft, Writing – review & editing. MB: Conceptualization, Formal analysis, Writing – original draft, Writing – review & editing. AO: Conceptualization, Supervision, Writing – original draft, Writing – review & editing.

## References

[1] Hales CM Fryar CD Carroll MD Freedman DS Ogden CL. Trends in obesity and severe obesity prevalence in US youth and adults by sex and age, 2007-2008 to 2015-2016. JAMA. (2018) 319:1723–5. doi: 10.1001/jama.2018.3060 PMC5876828 29570750

[2] World Health Organization. Obesity: preventing and managing the global epidemic. Report of a WHO consultation. World Health Organ Tech Rep Ser. (2000) 894:i–xii, 1–253. 11234459

[3] Lavie CJ Milani RV Ventura HO. Obesity and cardiovascular disease: risk factor, paradox, and impact of weight loss. J Am Coll Cardiol. (2009) 53:1925–32. doi: 10.1016/j.jacc.2008.12.068 19460605

[4] Vague J. The degree of masculine differentiation of obesities: a factor determining predisposition to diabetes, atherosclerosis, gout, and uric calculous disease. Am J Clin Nutr. (1956) 4:20–34. doi: 10.1093/ajcn/4.1.20 13282851

[5] Ibrahim MM. Subcutaneous and visceral adipose tissue: structural and functional differences. Obes Rev. (2010) 11:11–8. doi: 10.1111/j.1467-789X.2009.00623.x 19656312

[6] Pouliot MC Després JP Nadeau A Moorjani S Prud'Homme D Lupien PJ. Visceral obesity in men. Associations with glucose tolerance, plasma insulin, and lipoprotein levels. Diabetes. (1992) 41:826–34. doi: 10.2337/diab.41.7.826 1612197

[7] Neeland IJ Turer AT Ayers CR Powell-Wiley TM Vega GL Farzaneh-Far R. Dysfunctional adiposity and the risk of prediabetes and type 2 diabetes in obese adults. JAMA. (2012) 308:1150–9. doi: 10.1001/2012.jama.11132 PMC3556508 22990274

[8] Snijder MB Dekker JM Visser M Bouter LM Stehouwer CD Yudkin JS. Trunk fat and leg fat have independent and opposite associations with fasting and postload glucose levels: the Hoorn study. Diabetes Care. (2004) 27:372–7. doi: 10.2337/diacare.27.2.372 14747216

[9] Maislin G Ahmed MM Gooneratne N Thorne-Fitzgerald M Kim C Teff K. Single slice vs. volumetric MR assessment of visceral adipose tissue: reliability and validity among the overweight and obese. Obes (Silver Spring). (2012) 20:2124–32. doi: 10.1038/oby.2012.53 PMC3743719 22395811

[10] Irlbeck T Massaro JM Bamberg F O'Donnell CJ Hoffmann U Fox CS. Association between single-slice measurements of visceral and abdominal subcutaneous adipose tissue with volumetric measurements: the Framingham Heart Study. Int J Obes (Lond). (2010) 34:781–7. doi: 10.1038/ijo.2009.279 PMC2982778 20065971

[11] Siren R Eriksson JG Vanhanen H. Waist circumference a good indicator of future risk for type 2 diabetes and cardiovascular disease. BMC Public Health. (2012) 12:631. doi: 10.1186/1471-2458-12-631 22877354 PMC3490795

[12] Expert panel on detection, evaluation, and treatment of high blood cholesterol in adults. Executive summary of the third report of the national cholesterol education program (NCEP) expert panel on detection, evaluation, and treatment of high blood cholesterol in adults (Adult treatment panel III). JAMA. (2001) 285:2486–97. doi: 10.1001/jama.285.19.2486 11368702

[13] Lemieux I Pascot A Couillard C Lamarche B Tchernof A Alméras N. Hypertriglyceridemic waist: a marker of the atherogenic metabolic triad (hyperinsulinemia; hyperapolipoprotein B; small, dense LDL) in men? Circulation. (2000) 102:179–84. doi: 10.1161/01.CIR.102.2.179 10889128

[14] Amato MC Giordano C Galia M Criscimanna A Vitabile S Midiri M. Visceral Adiposity Index: a reliable indicator of visceral fat function associated with cardiometabolic risk. Diabetes Care. (2010) 33:920–2. doi: 10.2337/dc09-1825 PMC2845052 20067971

[15] Kahn HS. The "lipid accumulation product" performs better than the body mass index for recognizing cardiovascular risk: a population-based comparison. BMC Cardiovasc Disord. (2005) 5:26. doi: 10.1186/1471-2261-5-26 16150143 PMC1236917

[16] Krakauer NY Krakauer JC. A new body shape index predicts mortality hazard independently of body mass index. PloS One. (2012) 7:e39504. doi: 10.1371/journal.pone.0039504 22815707 PMC3399847

[17] Thomas DM Bredlau C Bosy-Westphal A Mueller M Shen W Gallagher D. Relationships between body roundness with body fat and visceral adipose tissue emerging from a new geometrical model. Obes (Silver Spring). (2013) 21:2264–71. doi: 10.1002/oby.20408 PMC3692604 23519954

[18] Bello-Chavolla OY Almeda-Valdes P Gomez-Velasco D Viveros-Ruiz T Cruz-Bautista I Romo-Romo A. METS-IR, a novel score to evaluate insulin sensitivity, is predictive of visceral adiposity and incident type 2 diabetes. Eur J Endocrinol. (2018) 178:533–44. doi: 10.1530/EJE-17-0883 29535168

[19] Bello-Chavolla OY Antonio-Villa NE Vargas-Vázquez A Viveros-Ruiz TL Almeda-Valdes P Gomez-Velasco D. Metabolic Score for Visceral Fat (METS-VF), a novel estimator of intra-abdominal fat content and cardio-metabolic health. Clin Nutr. (2020) 39:1613–21. doi: 10.1016/j.clnu.2019.07.012 31400997

[20] Zhu S Heymsfield SB Toyoshima H Wang Z Pietrobelli A Heshka S. Race-ethnicity-specific waist circumference cutoffs for identifying cardiovascular disease risk factors. Am J Clin Nutr. (2005) 81:409–15. doi: 10.1093/ajcn.81.2.409 15699228

[21] Kuddar Ç Çetin S. Investigation of affective traits affecting mathematics achievement by SEM and MARS methods. Int J Assess Tools Educ. (2022) 9:337–56. doi: 10.21449/ijate.982666

[22] Friedman JH. Multivariate adaptive regression splines. Ann Statist. (1991) 19:1–67. 10.1177/096228029500400303 8548103

[23] Onat A Uğur M Can G Yüksel H Hergenç G. Visceral adipose tissue and body fat mass: predictive values for and role of gender in cardiometabolic risk among Turks. Nutrition. (2010) 26:382–9. doi: 10.1016/j.nut.2009.05.019 19632090

[24] DeLong ER DeLong DM Clarke-Pearson DL. Comparing the areas under two or more correlated receiver operating characteristic curves: a nonparametric approach. Biometrics. (1988) 44:837–45. 3203132

[25] Pouliot MC Després JP Lemieux S Moorjani S Bouchard C Tremblay A. Waist circumference and abdominal sagittal diameter: best simple anthropometric indexes of abdominal visceral adipose tissue accumulation and related cardiovascular risk in men and women. Am J Cardiol. (1994) 73:460–8. doi: 10.1016/0002-9149(94)90676-9 8141087

[26] Flint AJ Rimm EB. Commentary: Obesity and cardiovascular disease risk among the young and old–is BMI the wrong benchmark? Int J Epidemiol. (2006) 35:187–9. doi: 10.1093/ije/dyi298 16394117

[27] Pischon T Boeing H Hoffmann K Bergmann M Schulze MB Overvad K. General and abdominal adiposity and risk of death in Europe [published correction appears. N Engl J Med. (2008) 359:2105–20. doi: 10.1056/NEJMoa0801891 19005195

[28] Weiss A Boaz M Beloosesky Y Kornowski R Grossman E. Body mass index and risk of all-cause and cardiovascular mortality in hospitalized elderly patients with diabetes mellitus. Diabetes Med. (2009) 26:253–9. doi: 10.1111/j.1464-5491.2009.02672.x 19317820

[29] Coutinho T Goel K Corrêa de Sá D Kragelund C Kanaya AM Zeller M. Central obesity and survival in subjects with coronary artery disease: a systematic review of the literature and collaborative analysis with individual subject data. J Am Coll Cardiol. (2011) 57:1877–86. doi: 10.1016/j.jacc.2010.11.058 21545944

[30] Manson JE Bassuk SS Hu FB Stampfer MJ Colditz GA Willett WC. Estimating the number of deaths due to obesity: can the divergent findings be reconciled? J Womens Health (Larchmt). (2007) 16:168–76. doi: 10.1089/jwh.2006.0080 17388733

[31] Borruel S Moltó JF Alpañés M Fernández-Durán E Álvarez-Blasco F Luque-Ramírez M. Surrogate markers of visceral adiposity in young adults: waist circumference and body mass index are more accurate than waist hip ratio, model of adipose distribution and visceral adiposity index. PloS One. (2014) 9:e114112. doi: 10.1371/journal.pone.0114112 25479351 PMC4257592

[32] Elisha B Messier V Karelis A Coderre L Bernard S Prud'homme D. The Visceral Adiposity Index: Relationship with cardiometabolic risk factors in obese and overweight postmenopausal women–a MONET group study. Appl Physiol Nutr Metab. (2013) 38:892–9. doi: 10.1139/apnm-2012-0307 23855278

[33] Ching YK Chin YS Appukutty M Gan WY Chan YM. Comparisons of conventional and novel anthropometric obesity indices to predict metabolic syndrome among vegetarians in Malaysia. Sci Rep. (2020) 10:20861. doi: 10.1038/s41598-020-78035-5 33257810 PMC7705716

[34] Yang J Wang F Wang J Han X Hu H Yu C. Using different anthropometric indices to assess prediction ability of type 2 diabetes in elderly population: a 5 year prospective study. BMC Geriatr. (2018) 18:218. doi: 10.1186/s12877-018-0912-2 30223783 PMC6142386

[35] Heine PA Taylor JA Iwamoto GA Lubahn DB Cooke PS. Increased adipose tissue in male and female estrogen receptor-alpha knockout mice. Proc Natl Acad Sci U S A. (2000) 97:12729–34. doi: 10.1073/pnas.97.23.12729 PMC18832 11070086

[36] Lovejoy JC Champagne CM de Jonge L Xie H Smith SR. Increased visceral fat and decreased energy expenditure during the menopausal transition. Int J Obes (Lond). (2008) 32:949–58. doi: 10.1038/ijo.2008.25 PMC2748330 18332882

[37] Lemieux S Prud'homme D Bouchard C Tremblay A Després JP. A single threshold value of waist girth identifies normal-weight and overweight subjects with excess visceral adipose tissue. Am J Clin Nutr. (1996) 64:685–93. doi: 10.1093/ajcn/64.5.685 8901786

[38] Kim JA Choi CJ Yum KS. Cut-off values of visceral fat area and waist circumference: diagnostic criteria for abdominal obesity in a Korean population. J Korean Med Sci. (2006) 21:1048–53. doi: 10.3346/jkms.2006.21.6.1048 PMC2721927 17179685

[39] Examination Committee of Criteria for 'Obesity Disease' in Japan; Japan Society for the Study of Obesity. New criteria for 'obesity disease' in Japan. Circ J. (2002) 66:987–92. doi: 10.1253/circj.66.987 12419927

